# Intraoperative radiation therapy for colon and rectal cancers: a clinical review

**DOI:** 10.1186/s13014-016-0752-1

**Published:** 2017-01-19

**Authors:** Michael G. Haddock

**Affiliations:** Department of Radiation Oncology, Mayo Clinic, 200 First St SW, Rochester, MN 55905 USA

**Keywords:** Colon cancer, Rectal cancer, IORT

## Abstract

Although there have been significant advances in the adjuvant therapy of colorectal cancer, results for patients have historically been poor when complete resection is unlikely or not possible. Similarly, locally recurrent colorectal cancer patients often experience significant tumor related morbidity and disease control and long term survival have historically been poor with standard therapies. Intraoperative radiation therapy (IORT) has been proposed as a possible tool for dose escalation in patients with locally advanced colorectal cancer.

For patients with locally advanced primary or recurrent colon cancer, the absence of prospective controlled trials limits the ability to draw definitive conclusions in completely resected patients. In subtotally resected patients, the available evidence is consistent with marked improvements in disease control and survival compared to historical controls. For patients with locally advanced primary or recurrent rectal cancer, a relatively large body of evidence suggests improved disease control and survival, especially in subtotally resected patients, with the addition of IORT to moderate dose external beam radiation (EBRT) and chemotherapy. The most important prognostic factor in nearly all series is the completeness of surgical resection. Many previously irradiated patients may be carefully re-treated with radiation and IORT in addition to chemotherapy resulting in long term survival in more than 25% of patients. Peripheral nerve is dose limiting for IORT and patients receiving 15 Gy or more are at higher risk.

IORT is a useful tool when dose escalation beyond EBRT tolerance limits is required for acceptable local control in patients with locally advanced primary or recurrent colorectal cancer. Previously irradiated patients should not be excluded from treatment consideration.

## Introduction

Despite the realized therapeutic gains, colorectal cancer remains the 5^th^ leading cause of cancer death in the world with more than 1 million new cases and more than 600,000 deaths per year [1]. Screening for colorectal cancer is an effective tool and where implemented has resulted in declines in cancer mortality [2]. When diagnosed at an early stage, the prognosis following surgical based treatment of colorectal cancer is excellent. However, for patients with locally advanced primary or recurrent colon cancer, often unresectable for cure, recurrence rates are high and survival is poor with conventional therapy [3]. Similarly, very locally advanced primary or recurrent rectal cancer patients have high rates of local and distant relapse and poor survival outcomes [4–6].

The concept of using radiation therapy during an operation to treat a malignancy originated more than 100 years ago and early investigations took place in Spain, Austria, Germany and the United States [7]. One of the earliest reports of the use of intraoperative radiation therapy (IORT) for treatment of rectal cancer using orthovoltage was reported by Stanford investigators in 1937 [8]. Investigation of IORT in the megavoltage era using high energy electrons or began in the 1980s with more than 100 reports on the use of IORT for colorectal cancer published in the last 20 years. The majority of reports are single institution retrospective series and very few prospective studies have been performed. This review will summarize the results of IORT investigations for treatment of primary or recurrent colorectal cancer in the megavoltage era.

### IORT rationale

Historically, radiation dose fractionation schemes used to treat colorectal cancer were based more on normal tissue tolerance doses than on consideration of biological effective dose needed to control tumor. In the abdomen and pelvis, small bowel is typically the dose limiting normal tissue. Doses exceeding 45–50 Gy in 1.8–2 Gy fractions to a significant volume of small bowel are associated with a greater than 5% risk of late stricture or ulceration [9]. QUANTEC guidelines suggest that not more than 195 cc of peritoneal space that may potentially contain small bowel receive greater than 45 Gy [10]. Doses of 45–50 Gy, standardly fractionated with concomitant 5-FU have been shown in multiple phase III trials to be effective in reducing the risk of local relapse when combined with R0 surgical resection [11, 12]. However, when chemoradiation is combined with an R1 or R2 resection for locally advanced primary or recurrent rectal cancer, 45–50 Gy has been largely ineffective in achieving local control.

Retrospective data suggest that doses well in excess of 50 Gy are required for control of microscopic or gross colorectal cancer. In the Mayo Clinic experience, doses of 40–60 Gy following subtotal resection of rectal cancer resulted in 70% local relapse in patients with microscopic residual disease and 86% local relapse in patients with gross residual disease [13]. In a small series from Massachusetts General Hospital there was evidence of a dose response in patients with subtotally resected rectal cancer. Following R1 resection, local failure was observed in 40% of patients who received 50–60 Gy, but only 11% in those who received > 60 Gy. For patients with gross residual disease, even doses of 60–70 Gy were not effective with local relapse observed in 67% of patients [14]. In a small prospective Mayo Clinic trial a dose of 50 Gy for unresectable, residual or recurrent colorectal cancer was associated with progression in the radiation field in 90% of patients [15]. Small bowel obstruction was observed in 16% of patients.

Modern prospective trials have confirmed the ineffectiveness of moderate dose radiation therapy to control residual colorectal cancer. In the MRC CR07 trial, a short course of 25 Gy in 5 fractions preoperatively was compared to surgical resection alone with postoperative chemoradiation delivered only to R1 resection patients. In the selective postoperative chemoradiation arm, the local relapse rate was 21% compared to 9% in the R0 patients treated with surgery alone [16]. In the Dutch TME randomized trial, patients in the surgery alone arm were treated with 50.4 Gy in 28 fractions postoperatively if they had a positive resection margin. Only 47% of the R1 resection patients received the protocol prescribed postoperative radiation (45–60 Gy). The local recurrence rate at 2-years was 17% in positive margin patients with radiation and 16% in those who did not receive radiation [17].

Because the volume of normal tissue included within an IORT field is small and because sensitive and dose limiting organs such as small intestine can be mobilized out of the IORT field, there is a strong rationale for investigating IORT as a means of increasing the effective radiation dose. In patients with locally advanced or recurrent colorectal cancer, IORT is considered when surgery alone results in unacceptable local control and an effective external beam dose in excess of 60–70 Gy is required for local control. IORT is delivered at the time of a planned operative procedure and is typically limited to patients without metastatic disease or patients with limited volume metastatic disease being treated with curative intent.

### IORT for colon cancer

Most patients with colon cancer are adequately treated with surgery with or without adjuvant systemic therapy. There is no established role for the routine use of radiation as an adjuvant therapy in colon cancer. An intergroup study (0130) evaluated the use of 5-fluorouracil (5-FU) and levamisole with or without adjuvant radiation in colon cancer patients following R0 resection. Eligible patients included those with tumor adherence or invasion of surrounding structures or those with T3N+ tumors of the ascending or descending colon. The radiation dose was 45–50.4 Gy in 1.8 Gy fractions. Although the study closed early with only 222 out of a planned 700 patients accrued and only 187 evaluable patients, the was no difference in survival, disease-free survival or local control with the addition of radiation therapy [18].

Although radiation therapy is not routinely indicated for colon cancer patients, there may be a role in patients with locally advanced disease that is not amenable or is unlikely to be amenable to R0 resection or in patients with local recurrence in retroperitoneal locations. Recurrent patients that may benefit include tumor bed recurrences as well as locally advanced nodal recurrences. In these groups of patients IORT has been investigated.

### Primary locally advanced colon cancer

IORT as a component of therapy for locally advanced colon cancer began to be investigated at Mayo Clinic in 1981. Schield et al. reported a series of 103 patients treated from 1974 to 1994, about half of whom were subtotally resected [3]. These patients were generally treated with postoperative external beam radiation (EBRT) with 5-FU. The median EBRT dose was 50.4 Gy in 28 fractions and all but 3 patients received between 40 and 60 Gy. Eleven patients, nine of whom were subtotally resected received IORT with a median dose of 15 Gy (range, 10–20 Gy). Results are summarized in Table 1. Resection margins were strongly correlated with disease relapse and survival. Escalation of the EBRT dose above 50 Gy was not associated with improved disease control or survival, but was associated with a 19% small bowel obstruction rate compared to 9% with 50 Gy or less (*p* = 0.08). Although the number of IORT patients was small, there was a statistically significant improvement in local control, distant control and survival in subtotally resected patients who received IORT in addition to EBRT.

**Table 1:** Disease control and survival in locally advanced colon cancer, Mayo Clinic results

Group	# Patients	5-year LR	5-year DM	5-year OS
R0 resection	50	10%	~30%	66%
R1 resection	18	54%	~57%	47%
R2 resection	35	79%	~68%	23%
		*p* < 0.0001	*p* = 0.002	*p* = 0.0009
EBRT > 50 Gy	73	36%	-	50%
EBRT ≤ 50 Gy	30	50%	-	45%
		*p* = 0.18		*p* = 0.16
R1-2 + IOERT	9	11%	~12%	76%
R1-2, no IOERT	44	82%	~76%	26%
		*p* = 0.02	*p* = 0.01	*p* = 0.04

Abbreviations: *EBRT* external beam radiation therapy, *LR* local relapse, *DM* distant metastases, *OS* overall survival, *IOERT* intraoperative electron radiation therapy

In a follow-up series from the Mayo Clinic, Mathis et al. reported results of multimodal therapy including IORT in 146 unresectable T4 colorectal cancers treated between 1981 and 2007 [6]. A subset of 40 patients had tumors located in the colon. EBRT was generally delivered preoperatively to a median dose of 50.4 Gy in 28 fractions. The median IORT dose was 12.5 Gy. Five year local control was 86% in the entire group and in the subset of 40 patients with colon cancer, the median survival was 7.2 years with a 5-years survival rate of 61%. Of note, in the entire group of patients, adjuvant chemotherapy with FOLFOX or FOLFIRI was associated with a 92% 5-year survival.

### Locally advanced recurrent colon cancer

Results of multimodality therapy including IORT for locally advanced recurrent colorectal cancer were reported in a Mayo Clinic series of 607 patients in 2011 [19]. This series included 180 patients with recurrent colon cancer treated between 1981 and 2008. EBRT was generally delivered preoperatively (median 45 Gy) with 5-FU and the median IORT dose was 15 Gy. About two thirds of the entire group of patients was subtotally resected and in this group central relapse within the IORT field was observed in only 16% of patients at 5 and 10 years. For the subset of 180 recurrent colon cancer patients, the observed 5-year survival was 34%.

### IORT for advanced regional nodal disease in colon cancer patients

Patients with locally advanced retroperitoneal nodal disease may represent a relatively favorable subset of colon cancer patients with treated with multimodality therapy. IORT as a component of therapy in colon cancer patients with advanced nodal disease has been evaluated in a Mayo Clinic series. Thirty-seven patients were treated between 1981 and 2000, prior to the modern chemotherapy era. Advanced nodal recurrence, defined as disease unlikely to be controlled with surgery alone, was present in 31 patients while 6 had advanced nodal disease at primary presentation. Patients were typically treated with 50.4 Gy in 28 fractions preoperatively with 5-FU. IORT was delivered with 9–15 MeV electrons to a median dose of 12.5 Gy. For the entire group, 5-year survival was 40% with 3-year local relapse in 14%, distant relapse in 36% and central relapse in the IORT field in only 7%. For the subset of patients without gross residual disease (R0 or R1 resection), the 5-year survival was 49% with a median survival of 53 months [20].

### IORT for rectal cancer

#### Primary locally advanced rectal cancer

Most patients with locally advanced rectal cancer are not likely to benefit from a dose escalation approach using IORT. In a pooled analysis of 5 North American adjuvant rectal cancer studies, standard chemoradiation was associated with a less than 10% risk of local relapse in patients with a single risk factor of T3N0 disease or T1-2 N1 disease [21]. Among patients with T4 node positive disease, however, local relapse was observed in 20–30% of patients treated with standard chemoradiation following R0 resection. The MERCURY study group reported a favorable subset of T3 rectal cancers based on MRI staging to include patients with less than 5 mm extramural spread, no abutment of mesorectal fascia and no extramural venous invasion or invasion of the intersphincteric plane for low tumors [21]. This group had a local recurrence rate of less than 2% without the use of any radiation therapy.

Selection of patients is a key factor for appropriate use of IORT in the primary setting. Patients who may benefit are those with T4 primaries in whom R0 resection is unlikely with surgery alone. The only modern prospective study of IORT for primary rectal cancer was conducted in patients unlikely to benefit from dose escalation. A French multicenter phase III trial conducted from 1993 to 2001 randomized patients treated with preoperative EBRT to IORT or observation at the time of resection [22]. Eligible patients were patients with T3 or T4 primary rectal cancer or node positive rectal cancer. Ninety percent of the patients on the study were T3 and 66% were node negative. The preoperative radiation dose was 40 Gy in 20 fractions and the IORT dose was 18 Gy. Local control at 5-years was observed in 93% of patients without IORT and 92% with IORT. There was no significant difference in distant relapse, disease-free survival, overall survival or toxicity between the treatment groups.

IORT may be appropriate for R0 resection patients when margins are close or when there has been response to preoperative chemoradiation that may leave margins at risk for harboring undetectable residual disease. Results of selected series of IORT for primary rectal cancer are presented in Table 2. With modern imaging techniques, patients at risk for potential subtotal resection should be identified preoperatively and treated with neoadjuvant chemoradiation. The most common regimen is 45–50 Gy in 1.8–2 Gy fractions with concomitant 5-FU or capecitabine. Surgery is typically done 4–8 weeks after the completion of radiation and the IORT dose has generally been in the range of 10–20 Gy. Local control in the EBRT field is above 85% in nearly all series. Distant relapse is reported in 25–50% of patients and reported 5-year survival ranges from 50–80%.

**Table 2:** Disease control and survival with IORT for locally advanced rectal cancer. Results of selected series

Study	# Patients	Years	EBRT dose, Gy	Margins	IORT dose, Gy	5-year LC	5-year DM	5-year OS
Willett, MGH [42]	20	1978–1989	50.4	R0	10–20	88%	-	53%^a^
Valentini, Rome [43]	29	1991–2006	45–55	R0	10–15	100%	-	-
Alberda, Rotterdam [44]	31	1996–2012	45–50^b^	R1	10^c^	84%	-	-
Zhang, Shanghai [45]	71	1994–2007	45–50.4	R0-1	10–20	90%	54%	75%
Sadahiro, Japan [46]	99	1991–2001	20	ns	15–25	98%	20%	79%
Mathis, Mayo Clinic [6]	106	1981–2007	50.4	R0-2	7.5–25	86%^d^	49%^d^	49%
Roeder, Heidelberg [47]	243	1991–2004	41.4	R0-2	10–15	92%	-	-
Sole, Madrid [23]	335	1995–2010	45–50.4	R0-1	10–15	92%	25%^e^	75%
Kusters, European pooled [24]	605	to 2005	45–50.4	R0-2	10–12.5	88%	29%	67%

Abbreviations: *IORT* intraoperative radiation therapy, *LC* local control, *DM* distant metastases, *OS* overall survival, *EBRT* external beam radiation therapy, *MGH* Massachusetts General Hospital

^a^Disease-free survival

^b^Some patients treated with 25 Gy in 5 fractions

^c^intraoperative high dose rate brachytherapy

^d^includes 40 colon primary patients

^e^crude

Several factors have been reported to be related to disease control and survival. The most consistently reported factor is the completeness of the surgical resection, a factor related to both surgical quality and biology. Even with the addition of IORT, a margin positive resection is associated with a 5-fold increase in the risk of locoregional relapse and increased risk of death [23, 24]. Despite this, local control is maintained in most patients with the addition of IORT even after R2 resection. In the MGH series the reported local control in patients with gross residual disease was 57% and in the Mayo Clinic series it was 73% [25, 26]. This is perhaps the strongest evidence suggesting a benefit for dose escalation with IORT.

Additional factors related to survival and disease control include timing of EBRT and chemotherapy use. Preoperative chemoradiation is preferred in the adjuvant treatment of rectal cancer based on improved local control and reduced toxicity [27]. In the Mayo IORT series, preoperative sequencing was also associated with improved survival (55% vs 38% 5-year) [27]. Because the predominant failure pattern in IORT series is distant relapse, effective systemic therapy is critical for survival improvements. The addition of 5-FU to EBRT was associated with a reduction in distant relapse (83% vs 41% 5-year) in the Mayo Clinic series and the administration of systemic adjuvant chemotherapy has been associated with improved survival in multiple series [6, 24, 26].

#### Recurrent rectal cancer

Although improvements in surgical technique (total mesorectal excision) and neoadjuvant therapy have significantly reduced the incidence of pelvic recurrence of rectal cancer, management of local recurrence remains problematic. Failure to control pelvic recurrence leads to pain, bleeding, urinary and rectal obstruction and can be the cause of death even in the absence of distant metastatic disease. With the possible exception of early anastomotic recurrences limited to the bowel wall that can be cured with resection alone, control of locally recurrent cancer requires multimodality therapy [28].

In an early Mayo Clinic experience, subtotal resection alone resulted in no 5-year survivors and the addition of moderate dose EBRT (median 50.4 Gy) resulted in 7% 5-year survival [4].

As is the case with primary rectal cancer, the most important factor associated with disease control and survival in IORT series is the completeness of surgical resection [19, 29–34]. Results of selected series including IORT for locally recurrent rectal cancer following R0, R1 and R2 resection are presented in Tables 3, 4 and 5. In patients in whom an RO resection can be accomplished, IORT is associated with local control in 60–80% of patients and 5-year survival in 40–50% of patients. When margins are microscopically positive, local control is in the range of 30–60% and 5-year survival is reported in 20–30%. In the case of gross residual disease, local control is in the range of 30–50% and 5-year survival is reported in 15–25%. Distant relapse is reported in more than 70% of patients if resection is macroscopically incomplete. Although there are no randomized comparisons, the results for incompletely resected patients appear to be an improvement over surgery with or without EBRT. In the Mayo Clinic series of palliative resection patients, 3-year survival was 44% and local relapse 40% in R2 patients who received IORT compared to 15% 3-year survival and 93% local relapse in non-IORT patients [4].

**Table 3:** Disease control and survival with IORT for locally recurrent rectal cancer in association with R0 resection. Results of selected series

Study	Years	# Patients	EBRT dose, Gy	IORT dose, Gy	IORT technique	5-year LC	5-year DM	5-year OS
Alektiar, MSKCC [30]	1992–1998	53	50.4^a^	10–18	IOHDR	43%	-	36%
Lindel, MGH [31]	1978–1997	25	50.4^b^	10–15	IOERT	56%	-	40%
Wiig, Norway [33]	1990–1999	18	46–50	15–20	IOERT	70%	-	60%
Eble, Heidelberg [33] ^c^	1991–1995	14	41.4	10–20	IOERT	79%	19%	71%^d^
Dresen, Eindhoven [34]	1994–2006	84	50.4^e^	10	IOERT	69%	34%	48%
Haddock, Mayo Clinic [19]	1981–2008	236	50.4^f^	12.5	IOERT	79%	48%	46%

Abbreviations: *R0* pathologically negative margins, *EBRT* external beam radiation therapy, *IORT* intraoperative radiation therapy, *LC* local control, *DM* distant metastases, *OS* overall survival, *MSKCC* Memorial Sloan Kettering Cancer Center, *MGH* Massachusetts General Hospital, *IOHDR* Intraoperative high dose rate brachytherapy

^a^50.4 in patients with no prior EBRT; no EBRT in patients with prior radiation

^b^20–50 Gy in previously irradiated patients

^c^4-year results

^d^4-year relapse free survival

^e^30.6 Gy in previously irradiated patients

^f^5–39.6 Gy in previously irradiated patients

**Table 4:** Disease control and survival with IORT for locally recurrent rectal cancer in association with R1 resection. Results of selected series

Study	Years	# Patients	EBRT dose, Gy	IORT dose, Gy	IORT technique	5-year LC	5-year DM	5-year OS
Alektiar, MSKCC [30]	1992–1998	21	50.4^a^	10–18	IOHDR	26%	-	11%
Wiig, Norway [33]	1990–1999	29	46–50	15–20	IOERT	50%	-	20%
Eble, Heidelberg [33]^b^	1991–1995	9	41.4	10–20	IOERT	67%	33%	33%^c^
Dresen, Eindhoven [34]^f^	1994–2006	34	50.4^d^	12.5	IOERT	29%	69%	27%
Haddock, Mayo Clinic [19]	1981–2008	224	50.4^e^	15	IOERT	56%	62%	27%

Abbreviations: *R1* microscopic residual disease, *EBRT* external beam radiation therapy, *IORT* intraoperative radiation therapy, *LC* local control, *DM* distant metastases, *OS* overall survival, *MSKCC* Memorial Sloan Kettering Cancer Center, *IOHDR* intraoperative high dose rate brachytherapy

^a^50.4 in patients with no prior EBRT; no EBRT in patients with prior radiation

^b^4-year results

^c^4-year relapse free survival

^d^30.6 Gy in previously irradiated patients

^e^5–39.6 Gy in previously irradiated patients

^f^3-year results

**Table 5:** Disease control and survival with IOERT for locally recurrent rectal cancer in association with R2 resection. Results of selected series

Study	Years	# Patients	EBRT dose, Gy	IORT dose, Gy	5-year LC	5-year DM	5-year OS
Lindel, MGH [31]	1978–1997	15	50.4^a^	15–20	12%	-	13%
Eble, Heidelberg [33]^b^	1991–1995	8	41.4	10–20	60%	75%	25%^c^
Dresen, Eindhoven [34]	1994–2006	29	50.4^d^	15–17.5	29%	71%	24%
Haddock, Mayo Clinic [19]	1981–2008	156	50.4^e^	20	49%	73%	16%

Abbreviations: *R2* gross residual disease, *EBRT* external beam radiation therapy, *IOERT* intraoperative electron radiation therapy, *LC* local control, *DM* distant metastases, *OS* overall survival, *MSKCC* Memorial Sloan Kettering Cancer Center, *MGH* Massachusetts General Hospital,*IOHDR* intraoperative high dose rate brachytherap

^a^20–50 Gy in previously irradiated patients

^b^4-year results

^c^4-year relapse free survival

^d^30.6 Gy in previously irradiated patients

^e^5-39.6 Gy in previously irradiated patients

#### Previously irradiated patients

A particularly challenging group of patients with locally recurrent rectal cancer are those who have previously received a course of radiation as adjuvant therapy for their primary malignancy or for another primary malignancy. Because of an increased risk of complications in these patients, the use of IORT without additional EBRT has been explored, but results have been poor. A Pamplona series reported the results of 10–20 Gy IORT in pelvic recurrences of rectal cancer with or without EBRT. Local control at 3-years was 0% with IORT alone and 30% with EBRT + IORT and survival improved from 12 to 38% with the addition of EBRT [35]. Similarly, a Lyon series reported on 50 patients treated with 10–25 Gy IORT with or without EBRT [36]. EBRT was withheld due to prior radiation or postoperative complications. Three year survival was 68% and local control 61% for the combination of EBRT and IORT versus 25% survival and 0% local control for IORT alone.

Despite the risk of late toxicity in previously irradiated patients, additional EBRT can be delivered to most patients with acceptable morbidity. In general, re-irradiation targets are limited to the gross tumor volume without inclusion of elective target volumes and with exclusion of all small bowel. In a University of Kentucky series, 103 previously irradiated rectal cancer patients received a 2^nd^ course of EBRT to a median dose of 34.8 Gy with 5-FU [37]. Only 34 of these patients also had a surgical resection. Although not insignificant, complications in 21% of patients including chronic severe diarrhea in 17% and small bowel obstruction in 15% were felt to be acceptable. A phase II study conducted in Rome included 59 previously irradiated recurrent rectal cancer patients who were treated with a second course of EBRT to a dose of 40.8 Gy in 1.2 Gy bid fractions with 5-FU [38]. In this series in which the target was limited to gross disease, late toxicity was mild with only one late small bowel obstruction.

Since the original publication of the University of Kentucky series [39], previously irradiated patients at Mayo Clinic have been re-irradiated to a dose of 30 Gy over 3 weeks with 5-FU immediately prior to surgery and IORT. Tumor volumes have been limited to gross disease with a small margin. Re-irradiation has been limited to the preoperative setting which allows much of the heavily irradiated volume to be removed. Although local relapse remains higher in previously irradiated patients (37% vs 22% at 5-years) [19], these results are much improved compared to results with IORT alone. Similarly, in the Eindhoven experience, improved results were noted in previously irradiated patients after the addition of preoperative EBRT to a dose of 30.6 Gy. With IORT alone, survival at 3-years was 25% and local control 38%. After the addition of re-irradiation, the 3-year survival improved to 48% and local control to 49% [34].

### IORT toxicity

Patients with very locally advanced primary and recurrent rectal cancer often experience significant tumor related and treatment related toxicity. Most treatment related effects are multifactorial and it is often difficult to attribute toxicity to a single modality. In a systematic review of 29 published studies including 3003 patients with locally advanced primary or recurrent colorectal cancer, IORT was associated with a significant improvement in local control and survival without an increase in total, urologic or anastomotic complications [40]. There was, however, an increased risk of wound complications following IORT. Wound infections and pelvic abscess are common complications reported in 25% or more of IORT patients in several series [30, 33, 34, 41]. In the Mayo Clinic series, the incidence of severe, life-threatening or fatal wound infection or abscess was 13% regardless of attribution with a rate of 7% potentially attributable to IORT [19].

With the addition of IORT to EBRT, the dose limiting normal tissue is typically peripheral nerve and neuropathy is the most commonly reported toxicity attributed to IORT in the pelvis. IORT related neuropathy most commonly manifests as pain without weakness or sensory loss. When it occurs, the pain is often chronic and may be severe, but often is manageable with gabapentin or pregabalin. Both the incidence and severity of IORT related neuropathy appear related to IORT dose. Even in previously irradiated patients, neuropathy incidence is related to IORT dose and not the total cumulative dose including EBRT. In the Mayo Clinic series, in the primary setting an IORT dose of 12.5 Gy or less was associated with a 3% incidence of grade 2 (requiring narcotics) or grade 3 (intractable pain) neuropathy while IORT doses of 15 Gy or higher were associated with a 23% incidence of grade 2–3 neuropathy [26]. In the locally recurrent disease setting, IORT doses of 12.5 Gy or less were associated with a 5% incidence of grade 2–3 neuropathy compared to 14% for IORT doses of 15 Gy or higher [19].

## Conclusions

Intraoperative radiation is a useful tool for dose escalation in patients with locally advanced primary and recurrent rectal cancer. It should be combined with preoperative EBRT and 5-FU or capecitabine. For patients with locally advanced primary or recurrent disease amenable to complete resection, the benefit of dose escalation with IORT should be prospectively evaluated. For subtotally resected patients, despite the lack of controlled trials, the body of available evidence strongly suggests that dose escalation with IORT in addition to EBRT and chemotherapy increases the likelihood of disease control and survival. Previously irradiated patients with local recurrence can often be safely re-irradiated and should receive preoperative EBRT with 5-FU or capecitabine. IORT doses of 15 Gy or higher appear to be associated with an increased risk of neuropathy. Distant relapse remains a significant challenge, but with the significant survival gains obtained with systemic therapy in the metastatic disease setting, long term control of pelvic disease has become even more important.

## Abbreviations

DM: Distant metastases
EBRT: External beam radiation therapy
IOERT: Intraoperative electron radiation therapy
IOHDR: Intraoperative high dose rate brachytherapy
IORT: Intraoperative radiation therapy
LC: Local control
MGH: Massachusetts General Hospital
MSKCC: Memorial Sloan Kettering Cancer Center
OS: Overall survival
